# Experimental investigation of sensitivity changes during encapsulation of piezoelectric composite materials

**DOI:** 10.1038/s41598-025-16973-8

**Published:** 2025-08-28

**Authors:** Rytis Mitkus, Vilius Kesminas, Markus Böl

**Affiliations:** Institute of Mechanics and Adaptronics, Technische Universität Braunschweig, D-38106 Braunschweig, Germany

**Keywords:** Piezoelectric composite sensors, Carbon fiber reinforced polymer (CFRP), Structural health monitoring (SHM), Sensor encapsulation, Engineering, Materials science

## Abstract

Piezoelectric (0-3) composites typically consist of a polymer matrix that contains piezoceramic particles. They can be used as sensors for structural health monitoring due to their lower acoustic impedance and ability to detect high-frequency waves. These composites have two thin electrodes on their surfaces, and cable connections that require electrical insulation. This insulation increases the durability of the sensor and provides additional protection. One way to achieve this is by encapsulating the sensor in polymer films. However, the sensitivity of the sensor may decrease due to an increase in overall stiffness after encapsulation, so this must also be evaluated. This study experimentally investigates and compares three different encapsulation designs with a non-encapsulated reference sample. The designs include (i) gluing and laminating the sensor onto a pre-prepared flexible printed circuit board, (ii) lamination of the sensor with polyethylene terephthalate, and (iii) lamination with polyetherimide. The sensitivity of the encapsulated sensors to low and high frequency vibrations was evaluated. The results show that an encapsulation sensor with adhesive tape and polyetherimide results in slightly lower sensitivity at lower frequencies, but almost no difference at higher frequencies. These results suggest that the proposed method is suitable for encapsulating sensors for use in structural health monitoring applications.

## Introduction

The growing demand for energy savings in the mechanical engineering industry has shifted the focus to the development of advanced lightweight structures^1–3^. These structures are being integrated into the automotive, aerospace, and other industries^1,2,4,5^. Fiber-reinforced composites, especially carbon fiber-reinforced polymers (CFRPs), have become relevant structural materials due to their high specific strength and stiffness. However, CFRP laminate structures have poor out-of-plane mechanical properties and are susceptible to impact damage, which can result in internal degradation^1–7^. Damage such as delamination between layers, fracture, and matrix cracks can impair the material’s properties and eventually lead to failure^1–4^. Delamination and cracking are particularly important issues because they cannot be visually detected^1,3,5,7,8^. Over time, this damage can accumulate and lead to the collapse or failure of the overall structure. To prevent critical conditions in lightweight structures, particularly those made of carbon or glass fiber-reinforced polymers, non-destructive evaluation (NDE) and structural health monitoring (SHM) are essential^3,5,8^.

SHM has emerged as a promising technique for assessing the condition of structures and accurately locating defects. It has attracted significant interest from the mechanical, electrical, and civil engineering communities^5,9,10^. One of SHM’s primary objectives is to detect damage and prevent failure in the early stages, thereby reducing costs by shortening inspection and maintenance cycles^5,11^. Although this process mainly uses sensors that are permanently attached to the structure to obtain performance information^1,4,5,9,12–14^, there are methods for measuring damage over distances^15^. Implemented structural sensing systems can evaluate structure’s condition over time by analyzing recorded data and design information^5,13,14^.

Systems for SHM based on guided ultrasonic waves (GUW), also known as Lamb waves, are widely used to monitor plate-like structures^16–19^. These waves are promising for monitoring lightweight CFRP structures because they can propagate over long distances, consume little energy, and experience low attenuation. Additionally, their small wavelength makes them sensitive to small defects^20^. Therefore, GUW systems are promising diagnostic tools^6,16,18,21,22^. Ultrasonic SHM methods typically involve contact-based techniques, such as piezoelectric transducers. These transducers require direct coupling with the structure using gels or adhesives to transmit and receive ultrasonic waves^23^. Piezoceramic transducers are popular in such systems for generating and detecting GUWs^16,18,21,24,25^ because they are effective, can produce high-frequency ultrasonic waves, and can be configured into arrays^26^, enabling inspections over large areas^14,27–29^. However, piezoceramics are brittle and heavy. They also have low damage strain and exhibit high acoustic impedance mismatch with plate-like CFRP structures. Therefore, piezoelectric polymers (e.g., polyvinylidene fluoride (PVDF)) and piezoelectric composite sensors (e.g., polymers filled with piezoceramic particles (PCS), also known as piezoelectric 0-3 composites^30–34^) are more suitable for GUW detection^18,35^. Note that a certain number of piezoceramic transducers are still needed in the SHM system to generate the GUWs (active SHM approach). Nevertheless, passive listening piezoelements can be replaced with piezoelectric polymers or composites instead of stiff, heavy piezoceramic transducers^17,21^. Piezoelectric actuators generate GUWs that propagate parallel to the structure’s surface. When a defect occurs, the wave energy is reflected and detected by the sensor network^4,16,24,25^. Contact-based SHM techniques offer high sensitivity and resolution, but they are limited by the need for physical contact^23^, making them impractical for large or complex structures^15^. Air-coupled ultrasonics is an alternative to contact-based techniques^36–41^ and has been investigated for over 30 years^42,43^. Air-coupled ultrasonics employs non-contact transducers that propagate waves through the air. This eliminates the need for coupling agents and enables the inspection of difficult-to-access areas. Additionally, it produces no secondary pollution^29,36,38,40,44^. However, air-coupled systems often face challenges related to lower signal strength and reduced resolution due to acoustic impedance mismatch in air, which makes them less effective at detecting fine defects than conventional ultrasonic methods^38,45,46^. Air-coupled ultrasonics are outside the scope of this study.

The acoustic impedance of PCS and piezoelectric polymers is closer to that of CFRP than to piezoceramic. The difference in acoustic impedance between materials determines how much of a sound wave is reflected or transmitted at the interface^47–49^. For SHM applications, the smallest acoustic impedance mismatch is required^50–52^. Acoustic impedance results from material density and ultrasonic velocity multiplication. Ultrasonic velocity depends on mechanical properties, such as Young’s modulus, Poisson’s ratio, and shear modulus. Density and mechanical properties can both be readily tailored during PCS manufacturing^53,54^, making PCS a more suitable candidate for SHM applications than piezoelectric polymers. Furthermore, depending on the polymer matrix used, piezoelectric ceramic composites can operate within a wider temperature range than PVDF sensors^55^. The reported PCS operate within the temperature range of $-70^{\circ }$C to $+200^{\circ }$C (limited by the conductive silver electrodes).

Surface mounting and embedding are two techniques used to integrate sensors into structures. Each technique has its own advantages and disadvantages^17,25,35,56,57^. Embedded sensors provide better protection and potentially stronger electromechanical coupling^24,35,58–60^, but they are more difficult to install and can compromise structural integrity^24,57,61^, leading to an undesirable reduction in the structure’s mechanical strength^24,57,62^. Surface-mounted sensors are easier to install and maintain, but they can be more susceptible to environmental influences^25^. Harsh environmental conditions, such as temperature, chemical exposure, and humidity, can limit the performance and reliability of surface-mounted sensors in various applications. Although PCS are less brittle than piezoceramics, they can still be relatively brittle and susceptible to damage from direct impacts. Furthermore, certain structures, such as CFRP, are conductive. This can pose challenges for piezoelectric sensors because the conductivity of the monitored structure can cause signal interference, affecting performance and reliability. To avoid this issue with conductive CFRP structures, a thin glass fiber layer can be used, though this complicates manufacturing and potentially alters the mechanical properties at the sensor position. Encapsulation serves multiple critical functions^63^: It provides electrical insulation to prevent short circuits and signal interference, it offers mechanical protection against fracture, delamination, and surface abrasion, and it shields the sensor from external environmental stressors, such as moisture, dust, temperature fluctuations, and chemical exposure. This improves long-term durability and reliability. In SHM and other applications, the performance and longevity of piezoceramic sensors depend on the quality and suitability of their encapsulation strategy. Therefore, encapsulating thin, relatively brittle piezoceramic composite sensors ensures electrical insulation, increases durability, and protects against external damage.

Many electronic components are filled with epoxy or silicone-based materials to protect them from environmental influences. While this approach is suitable for protecting certain thin sensors^64^, it is a relatively complex process that can result in an uneven sensor surface. Perfectly flat sensors provide better adhesion and signal detection. Additionally, piezoelectric transducers are almost always designed to be adhesively bonded to other structures^65^. There are multiple solutions for flat, encapsulated piezoceramics: Macro fiber composites (MFC, Smart Materials GmbH)^65–71^, active fiber composites (AFC, invented at the Massachusetts Institute of Technology)^66,72–77^, thermoplastic-compatible piezoceramic modules (TPMs) with piezoelectric fibers or plates^78–80^, other thin encapsulated piezoceramic layers^81,82^, flexible printed circuits with integrated piezoelectric transducers (SMART Layer, developed by Acellent Technologies)^83,84^, and piezoceramic covered by thin layers of ceramics^85,86^. These configurations differ in the materials and number of layers used, but they all have a thin, active piezoelectric element covered with thin, conductive metal layers, which are then covered with a thin plastic or ceramic film. These configurations use different piezoelectric components, such as plates, rods, or bars, and encapsulation for various purposes, including keeping rods or bars together or piezoceramic pre-stressing.

The SMART layer approach allows for the quick application of sensors to various substrates. When used as a full layer in the CFRP structure, this composition does not decrease mechanical properties or accelerate delamination, rather, it prevents delamination^83,84^. However, when used as a partial layer in larger structures, interlaminar stress concentrations form at the edges of the sensor-composite^87,88^. Regardless, embedding an object with different mechanical properties than CFRP between CFRP layers can cause delamination and local stress concentrations^11,89,90^.

An interesting approach was proposed for embedding thermoplastic-encapsulated sensors in a thermoplastic matrix CFRP structures^79,91^. This approach enables high strain transfer from the host structure to the sensors, as well as strong, homogeneous adhesion. However, thermoplastic composites typically require higher processing temperatures than thermoset epoxies, produce poorer fiber impregnation due to high melt viscosity^92^, and result in lower fiber-matrix adhesion and lower mechanical properties^93^. Additionally, the need to re-polarize the piezoceramic with a high voltage during or after composite formation adds an additional, complex step^79^.

A similar approach that results in a smaller reduction in mechanical properties and a smaller increase in delamination possibilities is to encapsulate each sensor individually without a large connecting element, such as a film. Encapsulating PCS with thin insulating polymer films is a simple and cost-effective approach^79,94^. Polyimide (PI), polyamide (PA), polyetherimide (PEI), and polyethylene (PET) are commonly used for sensor protection due to their high mechanical, thermal, and electrical properties^3,79,84,89,95–98^. However, these polymers’ different chemical structures can produce varying degrees of adhesion to the internal layers of sensors and host structures. Nevertheless, encapsulating sensors with films is simple and convenient because prefabricated films can be applied to flat sensors without special processes or equipment. If the electrodes can be deposited or patterned on the film prior to encapsulation, the process would be simplified, and the performance of the PCS would be minimally affected^97^. The encapsulation material and technique should minimally affect sensor sensitivity.

The main issue with encapsulating PCS is the reduction in strain transfer between the structure and the sensors. Encapsulating the sensor with any material reduces its sensitivity and overall performance^71,99^. Each additional layer of adhesive further reduces strain transfer. Researchers investigated the effect of encapsulating a seven-layer MFC^99^: Kapton, acrylic, electrode, piezoceramic fiber, epoxy composite, electrode, and acrylic. They found that encapsulation significantly decreases electromechanical coupling and piezoelectric stress coefficients. The stiffer the encapsulating materials are, the greater the increase in piezoelectric performance. However, changes in electrode thickness have a minimal (<1%) influence on overall transducer performance^71^. Encapsulating a PZT plate with low-temperature co-fired ceramic (LTCC) has been reported to reduce relative permittivity by up to 10%, reduce the hysteresis area, and decrease remnant polarization by approximately 21%^86^ due to mechanical clamping and chemical interactions between the matrix and the piezoceramic material. Researchers reported a very small reduction in electrical capacity (less than 4% over 122 days) after piezoelectric sensor lamination^80^. However, this reduction is likely due to the natural degradation of the piezoelectric material. Therefore, a reduction in PCS performance after encapsulation is expected.

The goal of this study is to test several methods of encapsulating PCS and evaluate the effect of encapsulation on sensitivity. We will compare the sensitivity of the presented encapsulation methods to low- and high-frequency surface vibrations.

## Material and methods

The detailed development and manufacturing methods of the PCS, used in this study, are described in Mitkus et al.^53^ and Mitkus^54^. Briefly, the PCS consists of 30 vol.% sodium potassium niobate piezoceramic powder (KNN, particle size $0.629~\pm ~0.226$ µm) from Nippon Chemical Industrial Co., Ltd., Tokyo, Japan, 0.2 wt.% multi-walled carbon nanotubes (COOH (carboxylic acid)-functionalized) from FutureCarbon GmbH, Bayreuth, Germany, 6 wt.% photo initiator (Diphenyl (2,4,6-trimethylbenzoyl)-phosphine Oxide (TPO), and photopolymer (High-Temperature V2, FLHTAM02 Formlabs, USA). All materials were dispersed in ethanol for a total of one hour using an ultrasonic sonotrode at either a 5 wt.% ceramic/solvent ratio or a 2 wt.% nanofiller/solvent ratio, depending on which required more ethanol. During dispersion, the suspensions’ temperature was kept below $50^{\circ }$C to minimize ethanol evaporation by surrounding the container with water and ice. Next, the suspension was stirred and heated overnight at $65^{\circ }$C to fully evaporate the ethanol. After ethanol evaporation, a castable, UV light-curable suspension is obtained.

The PCS manufactured in this study consists of two layers. First, a pre-cut polyvinyl chloride (PVC) film (Oracal 751C, 60 µm thick) was adhered to a glass plate. Then, a mold release agent (Safelease 30, AIRTECH Europe GmbH, Germany) was applied to the glass and PVC film in a single, thin layer using a sponge. The film was dried at room temperature for a few minutes using a cooling fan. The mold release agent decreases adhesion between the solidified composite and the glass/film. This improves the quality of the solidified composite and reduces residual stresses in the composite. The first layer of the PCS was tape-cast using a handheld metal blade, held at around $30^{\circ }$ in the prepared mold. The glass, along with the PVC film and the tape-cast composite suspension, was placed 50 mm below the UV light source (light wavelength = 405 nm, light intensity = 1.5 W/cm^2^ at a distance of 50 mm, EQ CL30 LED Flood, Henkel AG & Co. KGaA, D sseldorf, Germany). UV light was applied for 40 seconds from one side. After a brief cooling period, UV light was applied for another 40 seconds from the other side to achieve a homogeneous layer of the piezoelectric composite. Next, a second, thinner layer of PVC film (Oraguard 293, 30 µm thick) was glued on top of the previous PVC layer to create a mold for the second PCS layer. The second layer was then tape-cast and cured using a UV light source from one side for 80 seconds. While still in the mold, the PCS were cut to a final size of 20 $\times$ 20 mm using a laser (cleanCELL 1170, cleanLASER). After cutting, the samples were stored in the dark between two glass plates for several days to eliminate residual stresses. The average thickness of the produced PCS was 109 ± 17 µm.

The electrodes on the samples were manually shaped using conductive silver paint (CW2205, Chemtronics, USA) to ensure that both cable connections were on the same side and that one side of the sensor was completely flat. This was accomplished by extending the bottom electrode through one corner to the top side and wrapping it around so that the cables could be connected later. After forming the electrodes on both sides of the samples the samples were placed between two glass sheets covered with backing paper and allowed to dry at room temperature. Then, the samples were placed in an industrial furnace for the final curing process at $160^{\circ }$C for three hours. Next, the PCS were polarized with a direct current (DC) electric field of 15 kV/mm in silicone oil heated to $55^{\circ }$C for a total process time of 5 minutes (4 minute ramp-up to final conditions, 1 minute holding time at final conditions, instant ramp-down time). After polarization, oil residues were removed from the sensors with isopropyl alcohol. The typical properties of the manufactured, polarized, non-encapsulated PCS (hereafter referred to as the *reference sample*) are summarized in the Table 1.

**Table 1:** Properties of polarised, non-encapsulated PCS, manufactured at room temperature.

properties	unit	Value
thickness (without electrodes)	µm	$109~\pm ~17$
density^(i)^	g/cm^3^	2.15
Young’s modulus	GPa	$1.65~\pm ~0.13$
Poisson’s ratio^(ii)^	-	0.3
shear modulus^(i)^	GPa	636.9
capacitance^(iii)^	pF	$338.22~\pm ~37.90$
relative permittivity^(iii)^	-	$13.98~\pm ~0.55$
dielectric loss^(iii)^	-	$0.016~\pm ~0.0014$
piezoelectric charge coefficient $d_{31}$^(i)^	pC/N	$0.52~\pm ~0.13$
piezoelectric voltage coefficient $g_{31}$^(i)^	mV$\cdot$m/N	$4.02~\pm ~1.16$
electromechanical coupling coefficient $k_{31}$^(i)^	-	$0.00186~\pm ~0.000466$
sensitivity	mC/m^2^/m/m	$1.23~\pm ~0.24$
longitudinal wave velocity^(i)^	m/s	1017.7
shear wave velocity^(i)^	m/s	544.3

^(i)^ calculated.

^(ii)^ approximated because most polymers have Poisson’s ratio between 0.25 and 0.35^100^.

^(iii)^ value at 1 kHz.

The density

1 $$\begin{aligned} \rho _\textrm{PSC}=V_\textrm{Polymer}\cdot \rho _\textrm{Polymer}+V_\textrm{KNN}\cdot \rho _\textrm{KNN} \end{aligned}$$

of the PCS is determined by by $\rho _\textrm{Polymer}=1.14$ g/cm^3^ (High Temp Resin V2 (FLHTAM02), data sheet), $\rho _\textrm{KNN}=4.5$ g/cm^3^ (typical material density), and $V_\textrm{Polymer}=70$%, $V_\textrm{KNN}=30$%. In reality, the density should be slightly smaller due to the influence of 0.2 wt.% of MWCNTs and 6 wt.% of photoinitiator. These are not included in the calculations.

The Young’s modulus *E* is the average value of 56 specimens. Due to its change with PCS thickness (introduced by curing with UV light), Young’s modulus is calculated individually for every specimen using linear regression of the Young’s modulus values measured with specimens of varying thicknesses. For more detailed information, the reader is referred to Mitkus^54^.

The capacitance, relative permittivity, and dielectric loss were measured at 1 kHz, at least 24 hours after polarization, on 48 specimens.

The shear modulus

2 $$\begin{aligned} G=\dfrac{E}{2(1+\nu )} \end{aligned}$$

is calculated using the average Young’s modulus *E* and Poisson’s ratio $\nu$. The longitudinal wave velocity

3 $$\begin{aligned} v_l=\sqrt{\dfrac{E(1-\nu )}{\rho (1+\nu )(1-2\nu )}} \end{aligned}$$

is also calculated also using the average Young’s modulus. Finally, the shear wave velocity

4 $$\begin{aligned} v_s=\sqrt{\dfrac{G}{\rho }}. \end{aligned}$$

is calculated using the determined shear modulus and density.

The SHM performance of encapsulated PCS is evaluated by adhering the PCS to thin CFRP plates. The plates used in the experiments were manufactured using the vacuum-assisted resin transfer molding (VARTM) method. Four layers of unidirectional carbon fiber (Zoltek^TM^ PX35 50k, Fabric UD200, ZOLTEK Corporation) were stacked in a cross-ply configuration aligned with the fibers in the $90^{\circ }/0^{\circ }/0^{\circ }/90^{\circ }$ direction. During this process, resin is injected under vacuum into the stacked carbon fibers to minimize porosity and voids in the resulting CFRP composite. This method also ensures a nearly constant thickness throughout the composite laminate.

However, due to the nature of the manufacturing process, defects may occur at the edges of the structure, such as epoxy residue or misaligned carbon fibers. To ensure the precision of the CFRP, the edges were machined, resulting in a final CFRP plate dimension of 450 $\times$ 450 $\times$ 1.25 mm. One side of the plate was very smooth and used to glue the samples for the study, while the other side was rough due to the special mesh used in the manufacturing process.

The epoxy resin was made from RIMR426 resin and MGS RIMH435 hardener (Lange & Ritter GmbH, Germany). This combination was chosen for its relatively low viscosity, which allows for efficient impregnation of carbon fibers in the VARTM process, and for its 120-minute curing time. The recommended weight ratio of 100:26 (resin to hardener) was used. After mixing, the suspension was degassed in a vacuum chamber for 15 minutes to remove air bubbles, immediately prior to use in manufacturing CFRP. After infiltration, the CFRP plates were left under vacuum for 24 hours. Final curing took place in an industrial furnace, where the plates were heated to $80^{\circ }$C for 10 hours.

Three different encapsulation designs were fabricated, as well as a non-encapsulated reference sensor. Design 1 was inspired by commercially available piezoelectric SMART layers (e.g., Acellent^84^), in which the piezoelectric transducer is encapsulated on a prefabricated flexible printed circuit board (FPCB). In this study, however, the PCS are glued to the FPCB and laminated with a film on top. Designs 2 and 3 are based on simply laminating of PCS with different films. For this study, the PCS were laminated using a Maxdome laminator with standard hot lamination settings. Thin copper cables, 100 µm in diameter and coated with a thin layer of polyimide, were connected to the PCS with conductive silver ink (the same ink used to produce the PCS’s electrodes) before lamination.

**Fig. 1:**
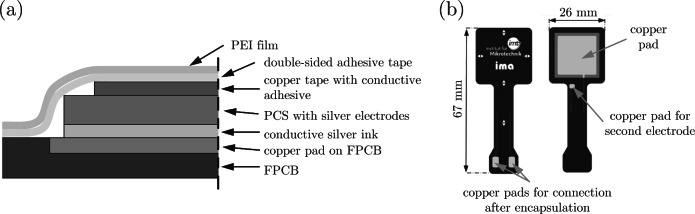
Illustration of design 1: (**a**) Cross-sectional view of the structural layout and (**b**) top view.

**Design 1:** The PCS was glued to the FPCB, which was then covered with 50 µm-thick ultra-high temperature, double-sided adhesive tape (9077, 3M) and 25 µm-thick PEI film (Goodfellow GmbH, Germany), as shown in Fig. 1 (a). The FPCB design is shown in (b). It was designed to be embedded in CFRP structures with the tail left outside for connection. There is also a small copper pad to extend the connection point for the second electrode connection as shown in Fig. 1 (b). SF305C (polyimide film, Shengyi Technology) and RF775 (polyimide film, Panasonic) were selected for produce the FPCB because they are thin and have good mechanical properties and can withstand relatively high temperature (up to $160^{\circ }$C). RF775 was used to apply the copper tracks and SF305C was used to encapsulate the RF775 on both sides. The FPCB has two copper layers and was manufactured by Multi Circuit Boards Ltd. The copper surfaces are electrolytically deposited rather than rolled to provide better ductility, cheaper production, and higher conductivity. First, the PCS with silver electrodes is glued to the surface of the FPCB with a thin layer of conductive silver ink (the same ink used for the electrodes). Next, precut copper foil with conductive adhesive is applied to connect the top layer of the PCS to the copper pad on the FPCB, see Fig. 1 (b). Then, a PEI film is attached with double-sided adhesive tape to complete the encapsulation. The entire sandwich structure is then passed through a hot laminator, and the excess PEI film is cut into the shape of the FPCB. The final dimensions of the square sensor part are 26 $\times$ 26 mm, and the overall thickness is 350 µm.

**Fig. 2:**
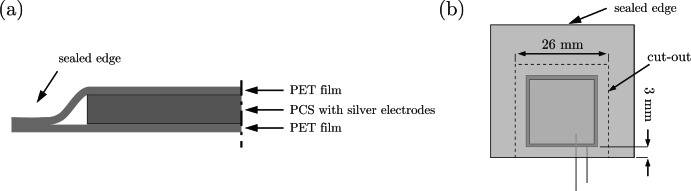
Schematic illustration of design 2: (**a**) Cross-sectional view of the structural layout and (**b**) top view.

**Design 2:** The PCS was laminated between two thin layers of PET film, a product also known as a PET laminating pouch, or film, with a thickness of 37.5 µm. This design was inspired by a commercial PVDF sensor (LDT0-028K). This design is the simplest to produce, and the PET films are inexpensive and widely available. No adhesive layers are required because the PET softens and adheres to itself and the PCS surfaces. As shown in Fig. 2 (a), the PCS was placed between two PET films and hot laminated with a laminator. The PET laminating pouch is already sealed on one side from the factory, which holds the sensor in place as it passes through the laminator. After lamination, the sensor is cut into a 26 $\times$ 26 mm square, see Fig. 2 (b). Note that the side with the protruding cables cannot be cut after lamination. Therefore, an additional 3 mm margin was left in advance to ensure sufficient connection between the layers after lamination. Thus, the PCS with the attached cables must be positioned before lamination, taking this into account. The thickness of this configuration is 185 µm.

**Fig. 3:**
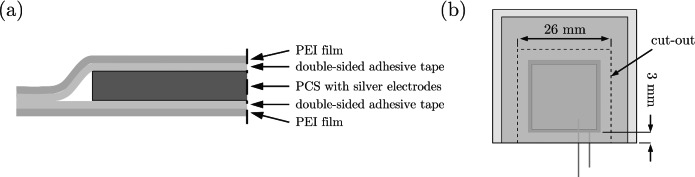
Schematic illustration of design 3: (**a**) Cross-sectional view of the structural layout and (**b**) top view.

**Design 3:** The PCS was laminated between two thin layers of PEI film. The PEI film was chosen because it adheres well to the CFRP matrix^101^. It has a higher softening temperature than PET, so it cannot be softened by a laminator and is not suitable for use with a laminator. Ultra-high temperature double-sided adhesive tape (9077, 50 µm thick, 3M) was used on both sides of the PCS between the PCS and the PEI film to ensure the tape seals the sensor. First, the adhesive tape was applied to the PEI film. Then, the PCS was placed on the tape, followed by the PEI film with the adhesive tape already applied. This created a sandwich structure, as shown in Fig. 3 (a). As with design 2, the side with the protruding cables could not be cut to size afterward. Therefore, a 3 mm margin was included to ensure sufficient bonding of the layers after lamination, see Fig. 3 (b). The other sides were cut to size after lamination. The final dimensions are 26 $\times$ 26 mm, and the total thickness is 260 µm. Table 2 summarizes the properties of the encapsulation materials used to help interpret the achieved results.

**Table 2:** Typical properties of encapsulation materials at room temperature.

parameter	unit	design 1_(RF775)_	design 2_(PET)_	design 3_(PEI)_
density	g/m^3^	1.4^(i)^	1.38	1.27
Young’s modulus	GPa	7.1	$2.8-3.1$	2.9
thickness	µm	25.0	37.5	25.0
single adhesive layer (if used)	µm	50.0	-	50.0
total thickness of encapsulated PCS	µm	350.0	185.0	260.0

^(i)^ assumed value since data can not be found.

**Fig. 4:**
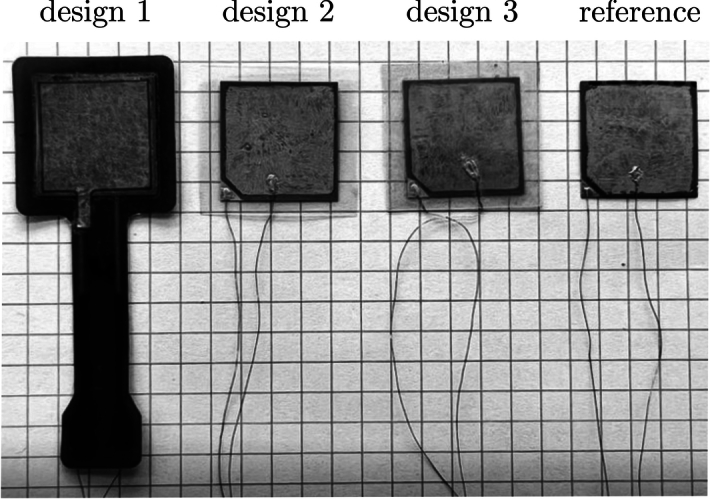
Piezoelectric composite sensors used in the study.

Finally, Fig. 4 shows samples of each design together with a reference sensor that is not encapsulated. The external dimensions of all of the encapsulated PCSs were 26 $\times$ 26 mm. Design 1 had an extended leg. The non-encapsulated sensor measured 20 $\times$ 20 mm.

A total of 62 non-encapsulated PCS composites were fabricated. Eight specimens were excluded due to failure during polarization or the presence of internal defects, such as invisible cracks. The composites were manufactured on two separate dates, approximately two weeks apart. Of the remaining 56 specimens, 48 were selected based on quality criteria, excluding those that were excessively thin, thick, or otherwise substandard. The 48 specimens were categorized into four groups of 12 according to their approximate thickness: Thin, below average, above average, and thick. Each group was then divided so that 3 specimens were laminated with design 1, 3 with design 2, 3 with design 3, and 3 were left unlaminated as reference specimens. This resulted in 12 specimens laminated with each design spanning the four thickness categories (3 specimens per thickness level). Of the three specimens in each thickness and design combination set, one was used for piezoelectric charge coefficient measurements, one was bonded to the plate described in the manuscript for testing, and one was tested on an alternative configuration plate not included in the current manuscript. Consequently, the results of 32 PCS specimens are reported in the manuscript. These include 16 specimens (4 designs $\times$ 4 thicknesses) tested under low-frequency conditions and 16 specimens (4 designs $\times$ 4 thicknesses) tested under high-frequency conditions.

**Fig. 5:**
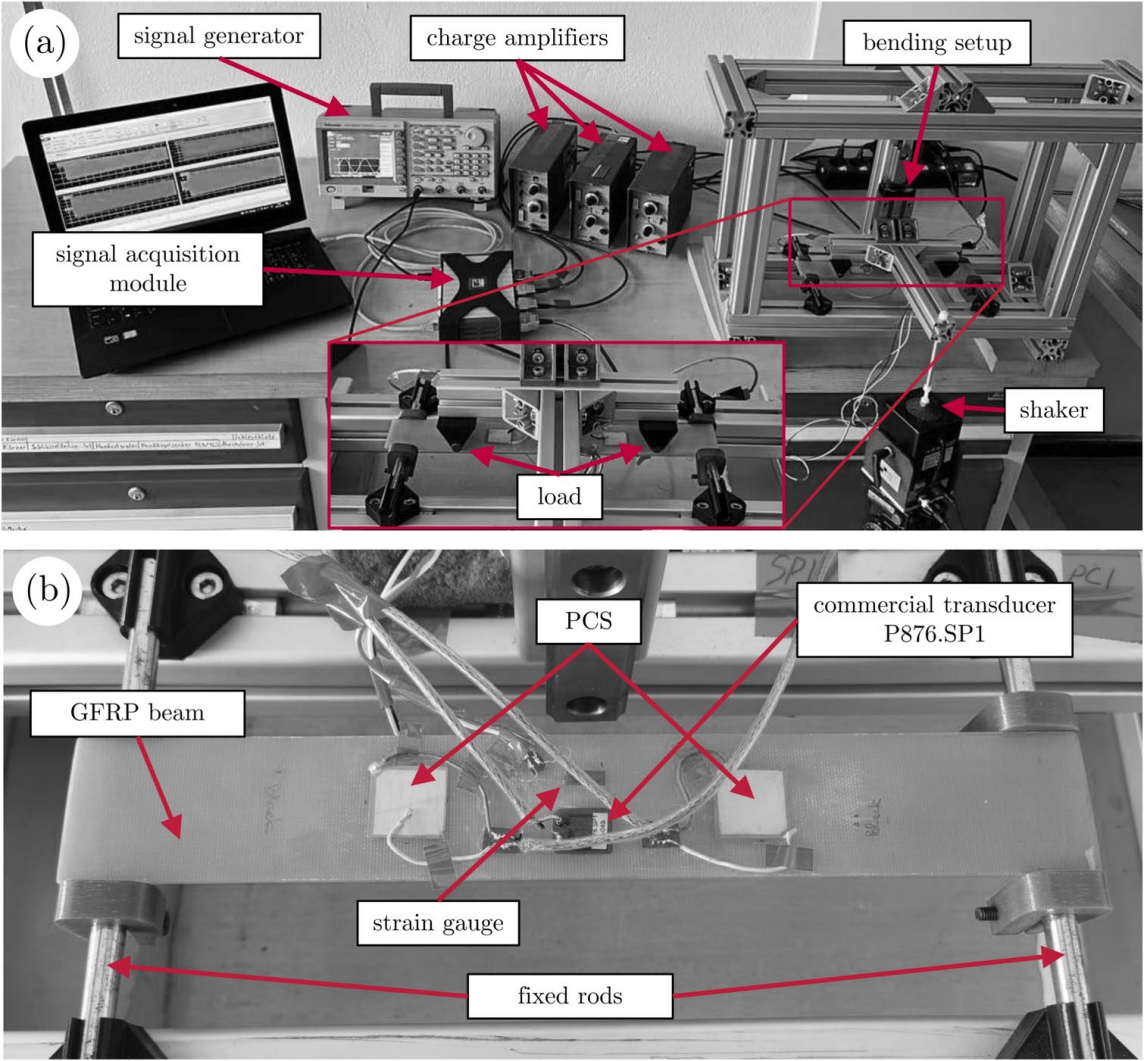
Setup of the four-point bending system used to measure piezoelectric charge coefficient $d_{31}$: (**a**) Equipment and configuration used, with loading and fixing points shown, and (**b**) GFRP beam with PCSs and commercial sensors attached.

The change in sensitivity with every encapsulation design at low frequencies was assessed by determining piezoelectric charge coefficient

5 $$\begin{aligned} d_{31}=\dfrac{1-\nu }{E}\,S_\textrm{sens}, \end{aligned}$$

by measuring the charge generated by the PCS and the strain applied to the beam. Note that the thickness of the adhesive and the influence of the sensor’s mechanical reinforcement on the beam are assumed to be negligible. In Equation (5), $\nu$ is the Poisson’s ratio, *E* denotes the Young’s modulus, and

6 $$\begin{aligned} S_\textrm{sens}=\dfrac{Q}{\varepsilon \,U} \end{aligned}$$

describes the sensitivity of the piezoelectric sensor. Herein, *Q* is the electric charge generated by the piezoelectric sensor, $\varepsilon$ describes the strain measured on the surface of the beam, and *U* is the PCS electrode area. Both electric charge and the strain on the surface of the beam were measured over time while exciting the beam to 22 Hz frequencies using a modified four-point bending system. This method was adapted and modified from^100^ and is described in earlier studies^54^. Briefly, the four-point bending system used is shown in Fig. 5 (a), where the entire frame is fixed to the table except for the two connected loading bars that can move up and down via an electric shaker. The system is designed to produce uniform strain on the surface of the substrate (a glass fiber-reinforced plastic (GFRP) beam with an isotropic layup, see Fig. 5 (b)) between the inner load points, see Fig. 5 (a), where the PCS and reference sensors are attached, see Fig. 5 (b). The 22 Hz frequency is the system’s first resonance frequency, ensuring relatively high bending strains (up to 250 µm/m) on the GFRP surface for PCS characterization. Two PCSs of interest are glued on each side of the beam. Note, that only one side of the GFRP beam is shown in Fig. 5 (b). In the center of each side of the beam, a commercial piezoceramic transducer (P-876.SP1, purchased from PI Ceramic, Germany) and two strain gauges (FLAB-6-17, manufactured by Tokyo Measuring Instruments Laboratory Co., Ltd., Japan) were glued.

In addition to the piezoelectric charge coefficient, the piezoelectric voltage coefficient

7 $$\begin{aligned} g_{31}=\dfrac{d_{31}}{\varepsilon _0\,\varepsilon _r}, \end{aligned}$$

was calculated, which depends on the charge coefficient $d_{31}$, the permittivity of a vacuum $\varepsilon _0$, and the relative permittivity

8 $$\begin{aligned} \varepsilon _r=\dfrac{C\,d}{\varepsilon _0\,U}. \end{aligned}$$

The relative permittivity is calculated from capacitance measurements taken at room temperature and 1 kHz using an LCR meter (LCR-300, Voltcraft). Finally, the thickness of the PCS is given by d.

The electromechanical coupling coefficient

9 $$\begin{aligned} k_{31}=\dfrac{d_{31}}{\sqrt{\varepsilon _0\varepsilon _r\dfrac{1}{E}}} \end{aligned}$$

describes the coupling between the electric field in the thickness direction and the mechanical strain in the plane direction. It was calculated from low-frequency measurements, taking into account the relative permittivity and Young’s modulus of each material.

A total of three specimens were used for each encapsulation method. Non-encapsulated PCS were used as a reference. To more accurately estimate the true value of the experiments, testing was conducted on each side of the beam five times. A total of ten experiments were conducted, with a single pair of PCS investigated each time.

**Fig. 6:**
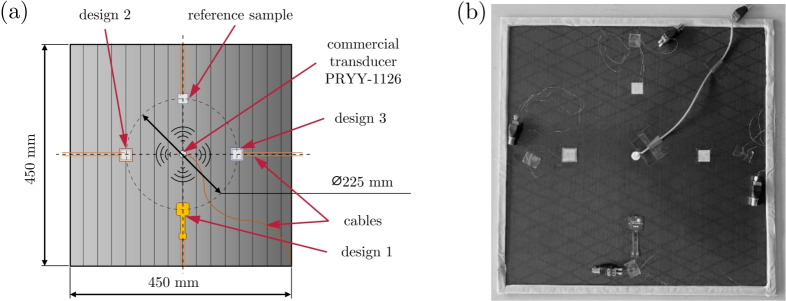
Performance test of the SHM measurement configuration: (**a**) Idealized illustration of the positioning of the central actuator and the PCS. The hatching illustrates the orientation of the CFRP fibers on the upper side, which has been varied by $90^{\circ }$, with each CFRP plate maintaining the PCS position constant. (**b**) Produced CFRP plate with glued-on PCS to perform the measurements.

The sensor’s SHM performance is determined using GUWs, which propagate in a thin CFRP plate at frequencies ranging from 5 to 250 kHz in 5 kHz intervals. The basic procedure and associated structure are described in detail in Roloff et al.^102^. In brief, the setup excites a wave field originating from the center of the CFRP plate. This wave field propagates uniformly and is captured by the PCS, which is glued at a specific distance around the central actuator, cf. Fig. 6. To generate a GUW within the plate, a commercial piezoceramic transducer (PRYY-1126, disc-shaped with wrap-around contact, purchased from PI Ceramics, Germany) was glued to the center of the CFRP plate. Both the commercial transducer and the PCS were attached using low viscosity cyanoacrylate adhesive. Similar adhesives demonstrated high performance in another study^103^. Considering the exceptionally smooth surfaces of the GFRP/CFRP and PCS substrates and the consistent application of a uniform load of ± 20 kg during bonding using a polished, flat metal block placed atop the PCS, the resulting adhesive layer thickness is expected to range between 10-30 µm. The reference specimen likely has a slightly thicker adhesive layer 20-50 µm due to the increased surface roughness associated with manually brushed conductive silver ink. Since all PCS samples in this study were bonded using the same adhesive and under identical conditions, the influence of the adhesive layer thickness is presumed to be negligible. This permits a qualitative comparison between different encapsulation designs. Although the generated wave field is assumed to have a concentric circular wavefront^102^, this is not entirely the case in reality due to the wrap-around contact. Nevertheless, to minimize possible irregularities in the wave field reaching the PCS, the cable and cable connection point at the transducer (wrap-around electrode) are positioned at $45^{\circ }$ angle from the plate’s edge. A shielded cable was used for the transducer to prevent interference.

The PCSs to be investigated are glued on the same side of the CFRP plate, 112.5 mm from the center, see Fig. 6 (a). The PCSs (design 1) are glued with PEI and adhesive tape facing downwards, creating a configuration similar to design 3 between the CFRP plate and the PCS. The reference PCS is not encapsulated and must be electrically insulated from the CFRP plate. Therefore, a single 0.25 mm thick layer of glass fiber was first glued to the CFRP plate, followed by gluing the PCS to the glass fiber.

**Fig. 7:**
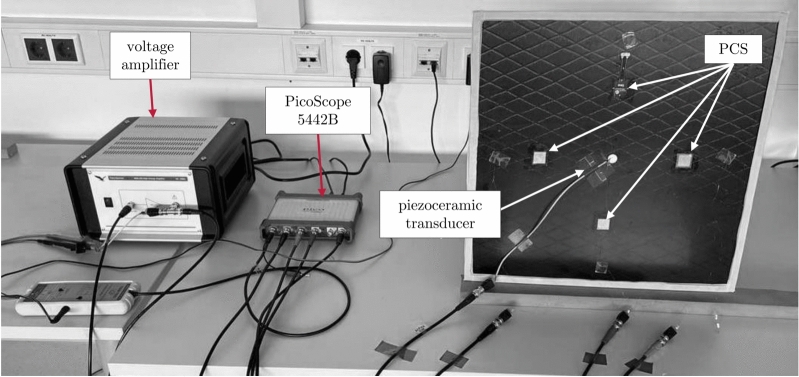
SHM performance testing setup.

A total of four CFRP plates were prepared. The positions of the encapsulated PCS were consistent across all plates, as illustrated in Fig. 6 (b). However, the commercial piezoceramic transducer (in the center) and the CFRP plate were rotated $90^{\circ }$ relative to each plate to minimize the influence of the layering of the CFRP plates and the rotation of the transducer on GUW generation. Soft vacuum-seal tape was attached to the edges of all plates to reduce reflections of propagating waves within the structure. During the SHM performance measurement, the CFRP plate was placed vertically on a thick layer of foam and leaned against a solid surface, see Fig. 7. A PicoScope 5442B was used as a signal acquisition unit and generator to provide an excitation signal to the actuator. The signal was amplified 50-fold using a high-voltage amplifier (WMA-300) and sent to the piezoceramic transducer, which was glued to the center of the plate. A five-cycle sine burst with a Hanning window was used for excitation as it produces a narrow-band signal with a low number of frequency components, similar to a Gaussian window^83^. Due to the short distance between the actuator and the PCS, it is difficult to distinguish between the $A_0$ and $S_0$ modes of Lamb waves. Thus, the peak-to-peak voltage was measured from the beginning of $S_0$ to the end of $A_0$^102^. Optimal excitation and reception of GUW by a piezoelectric transducer occurs when the sensor length *L* is approximately half the wavelength $\lambda$.

## Results and discussion

**Fig. 8:**
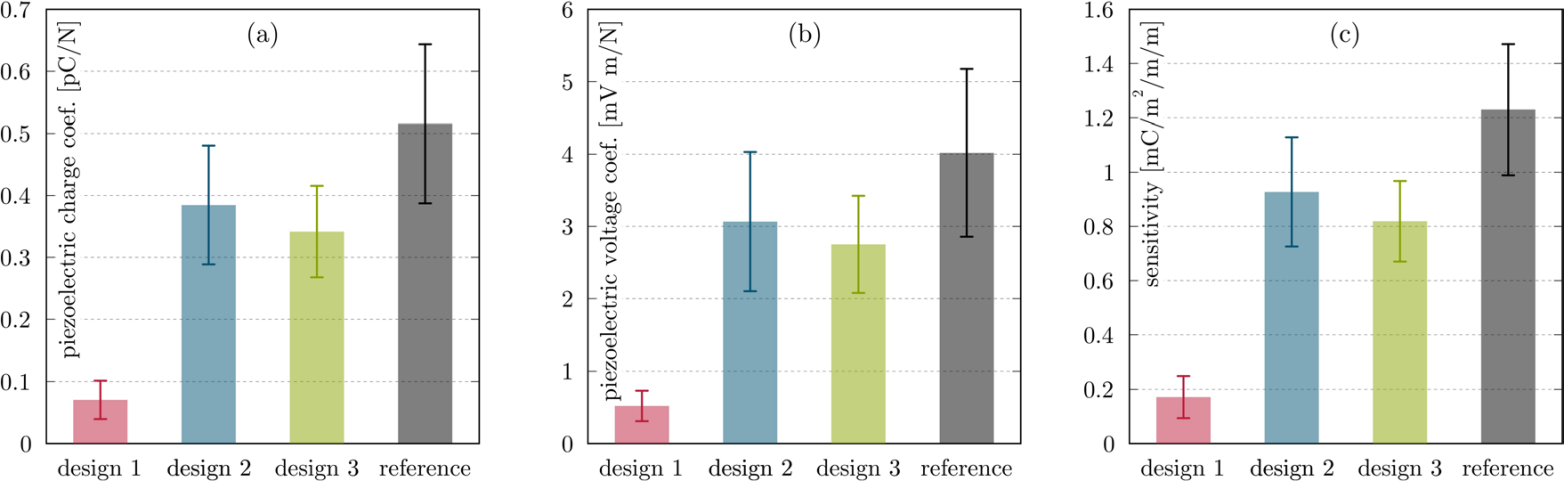
Piezoelectric performance of PCS: (**a**) Charge coefficients, (**b**) voltage coefficients, and (**c**) sensitivities.

Figure 8 shows the piezoelectric performance of the encapsulated PCS and the non-encapsulated reference sample. The determination of piezoelectric properties (e.g., $d_{31}$) was selected to assess sensitivity changes at low frequencies because this measurement directly reflects the material’s intrinsic electromechanical coupling efficiency, independent of system-level influences, such as electronics or environmental noise. Although SNR is relevant to the final performance of transducers in SHM applications, it depends heavily on the complete system configuration. In contrast, piezoelectric properties offer a consistent and comparable metric for evaluating the impact of encapsulation on the PCS itself. As expected, the reference sample exhibits the greatest piezoelectric performance at low frequencies. As shown in (a) and (b), encapsulation reduces the piezoelectric performance and, as shown in (c), the PCS sensitivity because it increases the sensor unit’s overall stiffness. This increased stiffness alters the distribution of mechanical strain when the sensor is stretched or bent. Consequently, less strain is transferred directly to the piezoelectric material, resulting in reduced PCS sensitivity. Furthermore, slight strain absorption can contribute to a damping effect. PCS laminated with thin PET or PEI films (design 2 and design 3 respectively), perform similarly, showing about 30% lower performance than the reference sample. Both designs have similar structural configurations because PET and PEI have comparable Young’s moduli and densities. However, design 3 incorporates an additional soft, nonwoven adhesive layer that may increase overall stiffness due to added thickness and enhance damping due to the compliant nature of the interfacial material between the adhesive layer and the PCS. Despite these potential influences, the similarity in performance observed between the two designs at low frequencies indicates that the effects of this additional stiffening and damping are minimal within this range. Of the evaluated designs, the thickest encapsulation configuration (design 1) has the lowest performance, with sensitivity reduced by more than seven times. This significant reduction is due to the additional copper adhesive layer between the CFRP beam and the PCS, as well as the stiffening effect of the FPCB carrier layer. Other researchers have conducted extensive simulation studies on MFC-type transducers (Kapton, Acrylic, electrode, piezoceramic fibre and epoxy composite, electrode, acrylic, Kapton) and found that the performance of piezoelectric transducers decreases with increasing electrode thickness^71,99^. Therefore, any material applied to the PCS (e.g., copper tape in design 1 and other films in other designs) increases the PCS’s overall stiffness and decreases its electromechanical coupling properties, thereby reinforcing the PCS.

Depending on the encapsulation design, the distance between the PCS and the substrate varies, excluding the adhesive layer thickness. Design 1 consists of copper tape, adhesive tape, and PEI foil, with a distance of 125 µm. Design 2 consists of PET laminate, with a distance of 37.5 µm. Design 3 consists of adhesive tape and PEI foil, with a distance of 75 µm. These distances do not allow any conclusions to be drawn about the measured performance. At first glance, the variation in the densities of the encapsulated PCS aligns with the observed results - decreasing the density of the encapsulated PCS leads to decreased performance. The encapsulation materials have lower densities ($\rho _\textrm{film}=1.3-1.5$ g/cm^3^) than the PCS ($\rho _\textrm{PCS} \approx 2.15$ g/cm^3^, see Table 1. However, density, which mainly influences acoustic impedance and mass/inertia, plays a more significant role in performance at higher frequencies and cannot be the main reason for this result. Additionally, the Young’s moduli of the encapsulation materials are higher than that of the PCS: $E_\textrm{film}=2.9-7.1$ GPa, see Table 2 and $E_\textrm{PCS}=1.654$ GPa. A precise comparison is complicated by the structural complexity of design 1; however, the higher Young’s modulus of the encapsulating layer appears to act as a mechanical constraint, limiting the deformation of the PCS. The increased Young’s modulus of the encapsulated PCS, together with the increased overall sensor thickness, is the main factor influencing decreased performance at low frequencies.

The standard deviation of the results is relatively high for most designs, except for design 1. The thick FPCB carrier layer in design 1 absorbs and dampens almost all of the strain induced during the test at the carrier surface where the sensor is attached, which explains this difference. This absorption results in significantly lower mechanical deformation of the PCS. Standard deviations appear to be a percentage of the total performance, with lower values corresponding to significantly lower standard deviations (e.g., design 1). Due to the significant overlap of standard deviations, no clear conclusions can be drawn about the relative performance of designs 2 and 3. Both designs perform about 30% worse than the reference PCS and have similar characteristics.

**Fig. 9:**
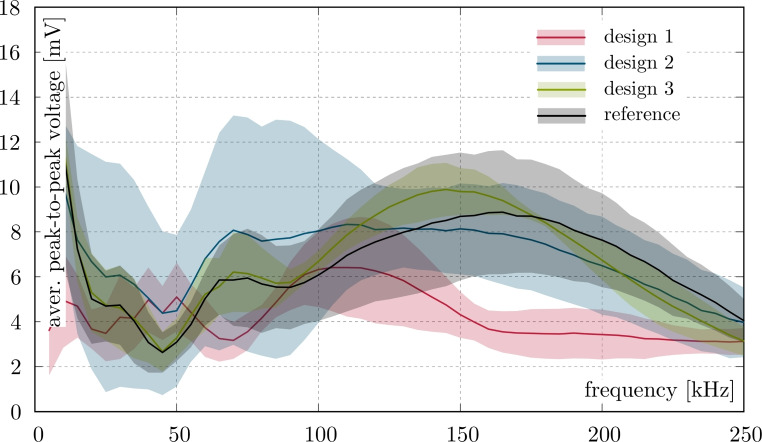
Average peak-to-peak voltages of surface bonded sensors.

**Figure 10:**
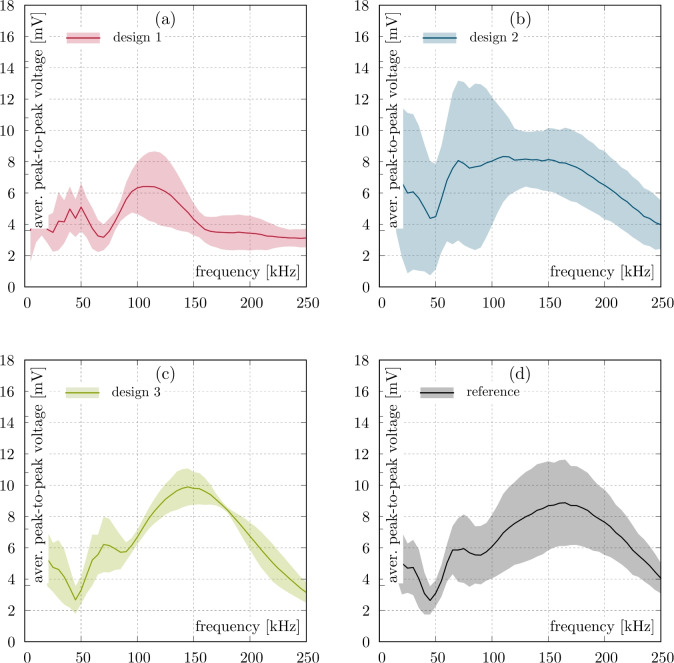
Average peak-to-peak voltages of surface glued sensors for (**a**) design 1, (**b**) design 2, (**c**) design 3, and (**d**) reference sample.

Figure 9 illustrates the sensitivity of each examined encapsulation method to GUW acquisition at various frequencies. For a better understanding, Figure 10 shows separate diagrams illustrating each design. All results are based on four measurements (four sensors of every design on four CFRP plates). The sensors qualitatively exhibit similar behavior, with maxima and minima occurring at certain frequencies. However, design 1 (PCS glued to FPCB and laminated with copper foil, adhesive tape, and PEI) exhibits different behavior. This configuration has low peak-to-peak voltage across the entire frequency bandwidth, particularly at higher frequencies. A peak in the measured peak-to-peak voltage is reached around 100-125 kHz, however, the overall measured values are low. Due to its nearly flat signal across the frequency range and low bandwidth compared to the other examined designs, this PCS encapsulation method is considered unsuitable for SHM measurements and will not be discussed further.

Overall, the maximum peak-to-peak voltages generated by the PCS are low. These voltages are measured directly from the PCS without amplification by charge amplifiers. The results presented here are similar to earlier reports of PCS behavior with similar maxima and minima frequencies when glued to an isotropic aluminum plate using the same test setup^102^.

Almost all of the measured samples show relatively high standard deviations that vary depending on the frequency. The exception is design 3, see Fig. 9. Design 3 demonstrates a consistently low standard deviation over the entire evaluated frequency range. Consisting of a PCS encapsulated in PEI with adhesive tape, design 3 exhibits the highest amplitude at higher frequencies (125-175 kHz), surpassing even the reference PCS. Furthermore, its sensitivity frequencies below 90 kHz is comparable to that of the reference sample. However, overlapping error bars prevent definitive conclusions about statistical significance. Due to the electrical conductivity of CFRP, a thin glass fiber layer was placed between the reference sensors and the CFRP plate which decreases the strain measured by the reference sample. Since design 3 is less sensitive than the reference sample at low frequencies (22 Hz), one might expect a corresponding reduction in sensitivity at higher frequencies. Incorporating a glass fiber layer beneath the reference PCS reduces measurable strain, and the reduction appears slightly greater than with design 3. This suggests that the encapsulation approach may offer a slight performance advantage over adding a glass fiber layer alone. Furthermore, the stiffening and damping effects of encapsulation become increasingly significant at higher frequencies due to the viscoelastic nature of the encapsulating materials and the shorter wavelengths of guided waves. These factors make the sensor more sensitive to material-induced constraints and energy losses. However, the comparable performance observed in design 3 suggests that the lamination materials are well-matched in terms of their mechanical and acoustic properties, which effectively mitigates the adverse effects of encapsulation.

Analysis of the frequencies corresponding to the peaks shows that the reference design has maximum sensitivity at slightly higher frequencies than the encapsulated PCS. Design 2 shows higher sensitivity at lower frequencies (20-125 kHz), but lower sensitivity at higher frequencies (>140 kHz). However, due to the large error bars, no clear conclusions can be drawn about the relative performance of design 2. At the lowest evaluated frequencies (5-10 kHz), all PCS configurations examined in this study, except for design 1, show relatively high peak-to-peak voltages. Composite sensors with distributed piezoelectric particles can effectively cover a larger part of the wavelength and thus avoid destructive interference that occurs at higher frequencies^104^.

Interpreting results at higher frequencies is difficult and requires further discussion. In a thin CFRP plate (less than 1.5 mm thick), the dominant wave modes are the asymmetric *A* and symmetric *S* Lamb waves. These waves propagate through a combination of longitudinal and shear motion, making them highly effective for SHM applications. Considering the material used (CFRP), its 1.25 mm thickness, and the tested frequencies (5-250 kHz), we assume that, apart from the predominantly excited $A_0$ and $S_0$, both $A_1$ and $S_1$ may appear at higher frequencies (>150 kHz). However, attenuation in CFRP is usually higher than in metals, so we expect the amplitudes of $A_1$ and $S_1$ to be negligble. The propagation velocity of the $A_0$ wave (the lowest-order antisymmetric mode) is comparatively low (approximately 1000 m/s at 50 kHz) and increases nonlinearly with frequency. In contrast, the propagation velocity of the $S_0$ wave (the lowest-order symmetric mode) remains relatively stable (approximately 5500 m/s) over the examined frequency range. These velocities depend on the structure and vary with factors such as dimensions, material properties, layer structure, thickness, and frequency.

All designs except design 1, which will not be discussed further here, show minimal sensitivity at approximately 50 kHz. This may be due to an intrinsic reduction in the PCS’s sensitivity since the $A_0$ mode wavelength at this frequency is approximately equal to the PCS length (20 mm at an $A_0$ mode velocity of 1000 m/s). As previously mentioned, a piezoelectric transducer optimally excites and receives GUW when the sensor length is approximately half the wavelength. Since the active PCS material is 20 mm long and the wavelength is also 20 mm, reception of GUW by the PCS should be minimal.

The smaller peak observed between 65 and 85 kHz could be related to the propagation of the $S_0$ mode wave. Assuming an $S_0$ mode velocity of 6000 m/s, this wave has a corresponding wavelength of approximately 80 mm. This wavelength is four times longer than the sensor’s length and can contribute to increased sensitivity. The peak sensitivity observed across all designs at 130-175 kHz may result from the combined influence of the $A_0$ and $S_0$ mode waves, which creates maximum sensitivity. At these frequencies, the $A_0$ mode wavelength is about 10 mm (assuming a velocity of 1500 m/s), and the $S_0$ mode wavelength is about 40 mm (assuming a velocity of 6000 m/s). The drop in peak-to-peak voltage at frequencies above 225 kHz is linked to the viscoelasticity of the polymer matrix because the stress and strain of viscoelastic materials are phase-shifted at high frequencies. This leads to higher energy loss and improved energy dissipation.

The exact reasons for this result are unclear and are most likely a combination of several factors. The thickness and density of the encapsulation materials and adhesives also affect sensor performance. However, accurately quantifying their individual effects is highly challenging. This is primarily due to the need for identical materials in varying thicknesses and numerous sensor configurations and experimental trials to isolate the impact of each varying parameter. The aim of this study is to qualitatively compare the performance of the PCS encapsulation method. All examined encapsulation designs except Design 1 yield similar results, suggesting GUW detection is possible, though further specialized research is needed. Design 2 shows high performance at lower frequencies and comparable performance at higher frequencies. Unfortunately, it also shows very high error bars, indicating possible low reliability of the configuration. Design 3 shows superior overall performance, characterized by high sensitivity and low standard deviation in measurements. Considering that the reference sample used for comparison was glued to a thin glass fiber layer, the encapsulated sensors’ GUW detection performance is somewhat lower. However, due to the conductivity of the CFRP plate, non-encapsulated PCSs always require an insulating layer. Therefore, design 3 seems to be practical for encapsulating PCSs and ensuring their high performance. Unfortunately, the available literature for direct comparison is limited. Most existing studies address the encapsulation of large-scale piezoelectric components for low-frequency energy harvesting applications in pavements^105^ or haptics and tactile sensing technologies^106–110^. Only one partially relevant study was identified, in which a piezoresistive (non-piezoelectric) MEMS transducer exhibited a 50% reduction in low-frequency sensitivity after being encapsulated with polydimethylsiloxane (PDMS)^109^.

## Conclusion

The main objective of this study was to qualitatively evaluate the effect of PCS encapsulation configurations on sensor performance at low and high frequencies. Design 1, in which the PCS is glued to the FPCB and laminated with copper film, adhesive tape, and PEI film, exhibited the poorest performance across all tested frequencies. This method is the most expensive and complex; the stiffening effect of its many layers likely contributes to its poor performance. In contrast, the two simpler, less expensive encapsulation designs exhibited slightly lower sensitivity than the non-encapsulated reference sensor at low frequencies (22 Hz) and nearly identical sensitivity to the reference sensor at higher frequencies (5-250 kHz). These results suggest that PCS encapsulation using thin polymer films and a simple hot lamination process is highly effective compared to non-encapsulated PCS.

## Data Availability

The data used and/or analyzed in this study are available from the corresponding author upon request.

## Appendix: Average peak-to-peak voltage of Lamb wave signals acquired by surface-bonded sensors

Figure 10 shows how sensitive each examined encapsulation method is to GUW acquisition at different frequencies. These graphs depict the individual components of the combined data shown in Figure 9 to provide a clearer understanding.
